# Advances in Antibody-Based Immune-Stimulating Drugs: Driving Innovation in Cancer Therapy

**DOI:** 10.3390/ijms26041440

**Published:** 2025-02-08

**Authors:** Ren-Jie Zhao, Xing-Xing Fan

**Affiliations:** Dr. Neher’s Biophysics Laboratory for Innovative Drug Discovery, State Key Laboratory of Quality Research in Chinese Medicine, Macau University of Science and Technology, Macau SAR 999078, China

**Keywords:** antibody-based immune-stimulating drugs, immune-stimulating antibody conjugates, bispecific antibodies, artificial intelligence

## Abstract

Antibody-based immune-stimulating drugs (ABIs) represent a transformative frontier in cancer immunotherapy, designed to reshape the tumor microenvironment and overcome immune suppression. This study highlighted recent advances in ABIs, including immune-stimulating antibody conjugates (ISACs), bispecific antibodies (BsAbs), and checkpoint blockade enhancers, with a focus on their mechanisms of action, clinical advancements, and challenges. Preclinical findings revealed that ISACs effectively boost overall anti-cancer immunity by reprogramming tumor-associated macrophages, enhancing T cell activation, and engaging other immune pathways. Similarly, BsAbs effectively redirect immune cells to tumors, achieving significant tumor regression. Additionally, artificial intelligence (AI) is revolutionizing the development of ABIs by optimizing drug design, identifying novel targets, and accelerating preclinical validation, enabling personalized therapeutic strategies. Despite these advancements, significant challenges remain, including immune resistance and off-target effects. Future research should prioritize next-generation multifunctional antibodies, AI-driven innovations, and combination therapies to enhance efficacy and expand therapeutic applications. Connecting these gaps could unlock the full potential of ABIs, upgrading cancer treatment and improving outcomes for patients with refractory or resistant tumors.

## 1. Introduction

In recent years, immunotherapy has revolutionized cancer treatment by leveraging the body’s immune system to combat tumors. Immune checkpoint inhibitors, such as those targeting PD-1/PD-L1 and CTLA-4 pathways, have achieved remarkable success in several malignancies. However, a substantial number of patients do not respond to these therapies due to the immunosuppressive nature of the tumor microenvironment (TME) [1]. This highlights the urgent need for therapeutic strategies that not only counteract immune suppression but also enhance immune effector functions.

The TME is a complex and dynamic ecosystem that plays a pivotal role in tumor immune evasion. Immune-suppressive cells, such as M2 macrophages, regulatory T cells (Tregs), and myeloid-derived suppressor cells (MDSCs), actively inhibit cytotoxic immune responses, facilitating tumor progression. In addition, chronic antigenic stimulation commonly leads to T cell exhaustion, in which the effector T cells lose the ability to effectively respond against the tumor [2]. This calls for novel therapeutic approaches capable of manipulating the immune microenvironment in a tumor-specific manner.

Multifunctional immune-stimulating drugs have emerged as promising tools in cancer immunotherapy. These therapies combine the tumor-targeting specificity of monoclonal antibodies with immune-stimulatory payloads, such as cytokines, Toll-like receptor agonists, and STING agonists, enabling a dual mechanism of action. For instance, immune-stimulating antibody conjugates (ISACs) deliver immune activators directly to the tumor site, thereby enhancing local immune responses while minimizing systemic toxicity [3]. Similarly, the bispecific antibodies engineered to target tumor antigens and immune effector cells simultaneously have also demonstrated synergistic potential in amplifying anti-tumor activity [4].

Integration of artificial intelligence (AI) into drug discovery accelerates antibody-based therapies by allowing the rapid identification of therapeutic targets, optimization of antibody structure, and predictions of drug interactions within the TME. Moreover, AI algorithms model complex immune dynamics that may guide novel therapy designs and optimize drug combinations. The latest advances in AI-driven drug design have already shown great potential in producing bispecific antibodies and immune-stimulatory conjugates targeting immune-suppressive cells and reinvigorating exhausted T cells [5].

This review provides an overview of recent advancements in antibody-based immune-activating agents for tumor treatment, with a particular focus on strategies aimed at neutralizing immune suppression within TME. Furthermore, it explores how AI is changing these therapies and gives an overview of how such innovations could be integrated into personalized cancer immunotherapy.

## 2. Unresolved Challenges in Tumor Immunotherapy

The TME is a complex and dynamic ecosystem that poses significant challenges to immunotherapy due to immune suppression, metabolic barriers, and structural obstacles that impair immune responses and facilitate tumor progression. The challenges can be summarized into three key areas as below: immunosuppressive cell populations, T cell exhaustion, and metabolic barriers. Improving innovative strategies to address these factors is critical to restoring immune function and enhancing the efficacy of cancer immunotherapies.

### 2.1. Accumulation of Diverse Immunosuppressive Cells in TME

Immunosuppressive cells within TME, including tumor-associated macrophages (TAMs), regulatory T cells (Tregs), and myeloid-derived suppressor cells (MDSCs), play a pivotal role in creating an immunosuppressive milieu that hinders anti-tumor immune responses and promotes tumor progression.

#### 2.1.1. M2 Macrophages

The TAMs in the TME predominantly exhibit an M2-like polarized phenotype, which is immunosuppressive, in contrast to the pro-inflammatory and anti-tumorigenic M1 phenotype [6,7]. This polarization is primarily driven by cytokines such as IL-10 and TGF-β, secreted by tumor and stromal cells, redirecting macrophage function away from initiating anti-tumor immune responses [8]. M2 TAMs significantly suppress immune responses by secreting immunosuppressive factors, including VEGF, prostaglandins, and matrix metalloproteinases (MMPs), which collectively support angiogenesis, extracellular matrix remodeling, and tumor metastasis [9]. These activities create a tumor-supportive environment while inhibiting cytotoxic immune cells such as T cells and natural killer cells [10]. Furthermore, M2 TAMs enhance immune evasion by recruiting Tregs and other suppressive immune cells, while also facilitating the expression of immune checkpoint molecules like PD-L1 on tumor and immune cells, thereby undermining the efficacy of immune checkpoint inhibitors and compromising immunotherapy outcomes [11,12].

Therapeutic resistance is another major challenge associated with M2 macrophages. The immune-cold environment fostered by these cells suppresses robust anti-tumor responses, reducing the effectiveness of immunotherapies. Furthermore, their secretion of growth factors like EGF and TGF-β enhances tumor cell proliferation and metastasis, both of which contribute to therapy resistance [13]. Addressing the challenges posed by M2 TAMs requires innovative therapeutic strategies. One strategy is the reprogramming of M2 TAMs into the M1 phenotype to restore their anti-tumor immune response. Agents targeting specific surface markers on M2 macrophages, such as CD206 or CCR2, are under investigation for their potential to selectively deplete these cells from the TME [6]. Another promising strategy involves the use of nanomedicine approaches, which deliver active therapeutic agents directly to the TAMs, thus minimizing off-target effects while maximizing efficacy [14]. Although these approaches show promise in improving cancer immunotherapy, they remain in the early stages of development.

#### 2.1.2. Tregs

Tregs perform an essential role in maintaining immune homeostasis through the suppression of excessive immune responses. In the TME, however, Tregs are often co-opted to facilitate tumor immune evasion. They inhibit anti-tumor immunity by suppressing the function of cytotoxic T lymphocytes (CTLs) and NK cells, efficiently neutralizing the immune system’s ability to target and eliminate cancer cells [15,16]. Tregs also secrete immunosuppressive cytokines, including IL-10 and TGF-β, which dampen the activity of effector immune cells and facilitate tumor growth [17]. Consequently, high infiltration in the TME by Tregs is associated with poor prognosis in many cancers. Tumors enriched with Tregs create an immunosuppressive environment that impairs immune cell activity and facilitates metastasis [18]. Furthermore, Tregs express high levels of immune checkpoint molecules such as CTLA-4 and PD-1, which further inhibit effector T cell activity and limit the efficacy of immune checkpoint inhibitors [19].

#### 2.1.3. MDSCs

MDSCs are another key immunosuppressive cell in the TME, playing a critical role in promoting tumor progression and enabling immune evasion. MDSCs exert their effects through the production of immunosuppressive molecules, including arginase-1 (ARG1), reactive oxygen species (ROS), and nitric oxide (NO), which directly impair cytotoxic immune cell function [20].

However, targeting MDSCs therapeutically presents significant challenges due to their heterogeneity and dynamic plasticity. MDSCs comprise polymorphonuclear and monocytic subsets, each with distinct suppressive mechanisms that further complicate therapeutic approaches. Emerging strategies to overcome these challenges include targeting specific signaling pathways, such as STAT3 and NF-κB, crucial for MDSC expansion and function. Additionally, combining MDSC-targeted therapies with immune checkpoint inhibitors holds potential for restoring effective anti-tumor immunity [21].

### 2.2. T Cell Exhaustion

Sustained antigen stimulation within TME, combined with immunosuppressive signals and metabolic stress, drives T cell exhaustion which is characterized by reduced effector function, diminished cytokine production, and the upregulation of inhibitory receptors such as PD-1, TIM-3, and LAG-3. Exhausted T cells fail to respond to tumor antigens, undermining the efficacy of immunotherapies [22]. Additionally, the immunosuppressive TME further accelerates T cell exhaustion and undermines the efficacy of clinical efficacy. For example, tumor cells, stromal cells, and regulatory immune cells such as Tregs and MDSCs can directly inhibit T cell function [23]. Hypoxia and metabolic dysregulation within the TME also contribute to exhaustion by limiting nutrient availability and enhancing oxidative stress on T cells [24]. Though anti-PD-1 antibodies are able to reinvigorate exhausted T cells, their effectiveness is greatly limited once the exhaustion becomes irreversible or if there is a predominance of other inhibitory pathways different from PD-1 [25]. Therefore, strategies to rejuvenate exhausted T cells are a focus in advancing immunotherapy.

### 2.3. Metabolic Challenges

#### 2.3.1. Nutrient Depletion

Nutrient depletion resulting from metabolic competition in the TME represents a major challenge for cancer immunotherapy. Tumor cells exhibit increased glucose and glutamine metabolism through aerobic glycolysis (Warburg effect) and glutaminolysis to maintain their rapid growth and proliferation. This excessive consumption depletes essential nutrients, creating a resource-scarce environment that hampers T cell activation, proliferation, and effector functions [26]. Insufficient glucose impairs glycolysis necessary for cytotoxic activities in effector T cells, whereas glutamine starvation reduces the biosynthesis of amino acids, nucleotides, and other important metabolites required for cell proliferation [27]. Addressing these metabolic constraints is vital for enhancing the efficacy of immunotherapies.

#### 2.3.2. Lactate Accumulation

Tumor cells predominantly rely on glycolysis, even under normoxic conditions, leading to excessive lactate production. This metabolic byproduct acidifies the TME, impairing immune cell functionality and further exacerbating the immunosuppressive environment [28]. In addition, lactate promotes polarization of macrophages toward the immunosuppressive M2 phenotype and inhibits the differentiation of monocytes into dendritic cells, reducing antigen presentation and weakening anti-tumor immunity [29]. This has made the targeting of lactate metabolism an attractive strategy, either by inhibiting its production by glycolysis inhibitors or enhancing its clearance by lactate transport inhibitors. Combined with approaches to alleviate nutrient competition, such strategies could significantly enhance the efficacy of immunotherapies.

#### 2.3.3. Arginine and Tryptophan Depletion

Enzymes such as arginase-1 and indoleamine 2,3-dioxygenase (IDO) deplete arginine and tryptophan in the TME, which are critical for T cell activation and survival, introducing additional metabolic barriers that further undermine immune responses. Arginine depletion impairs T cell receptor signaling and diminishes expression of the important T cell receptor complex chain CD3ζ. Simultaneously, tryptophan depletion leads to immunosuppressive metabolites such as kynurenine. All these alterations dampen effector T cell functions and lead to the differentiation and proliferation of Tregs, which further contribute to immunosuppression within the TME [30]. The therapeutic strategies targeting arginase-1 or IDO can restore amino acid levels, thus reinvigorating T cell function.

In summary, the TME imposes multiple metabolic and immunosuppressive barriers that hinder effective anti-tumor immunity, including nutrient depletion, lactate accumulation, and enzymatic amino acid degradation. These challenges underscore the importance of developing innovative strategies to restore immune cell functionality and counteract immunosuppression. Beyond metabolic interventions, multifunctional immune-stimulating drugs have emerged as a powerful class of therapeutics, offering the potential to directly enhance immune responses and overcome the suppressive mechanisms of the TME.

## 3. Antibody-Based Immune-Stimulating Drugs

**Figure 1:**
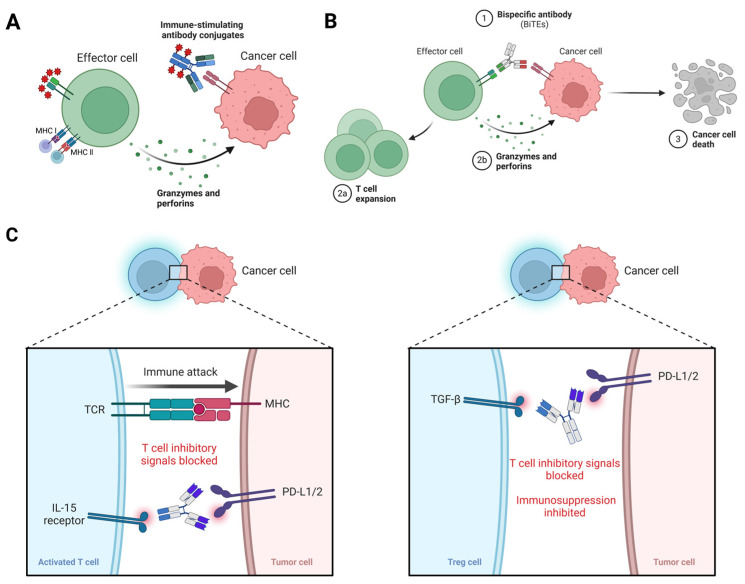
Mechanisms of action of ABIs. (**A**) ISACs combine tumor-specific antibodies with immune-stimulatory payloads, enabling localized immune activation within TME; (**B**) BsAbs connect T cells to tumor cells, facilitating tumor lysis; (**C**) immune checkpoint inhibitor against tumor cell.

ABIs have emerged as a promising therapeutic modality in cancer immunotherapy. These agents offer tumor-specific targeting and mechanisms to activate the immune system, overcoming immunosuppressive barriers within the TME (Figure 1). ABIs offer unique advantages, including precise delivery, reduced systemic toxicity, and enhanced innate and adaptive immune responses. Key innovations include ISACs, bispecific antibodies (BsAbs), and checkpoint blockade enhancers, each providing unique benefits.

### 3.1. ISACs

ISACs are a type of therapeutic antibody conjugated with immune-stimulatory payloads, such as TLR (Toll-like receptor) agonists or STING (stimulator of interferon genes) agonists, designed to enhance the immune system’s ability to recognize and fight tumors. They are engineered to combine tumor-specific antibodies with immune-stimulatory payloads, enabling localized immune activation within TME. This approach minimizes systemic toxicity while boosting anti-tumor immunity [31].

ISACs also serve as delivery systems for immune-stimulatory payloads, such as cytokines, TLR agonists, and STING agonists. These payloads reprogram the TME, converting an immunosuppressive milieu into an immune-active one. This dual function of tumor specificity and immune activation enhances both innate and adaptive immunity [32]. For instance, ISACs bearing TLR7/8/9 agonists activate dendritic cells, enhancing antigen presentation to T cells and, as a consequence, the generation of potent tumor-specific cytotoxic T cell responses, which is required for long-term control of the tumor. Antibodies conjugated with STING agonists further enhance innate immunity through the induction of type I interferons, enhancing anti-tumor responses [33,34,35]. All these cascade into higher adaptive immune response, leading to increased cytotoxic activity and tumor regression seen in preclinical studies. Similarly, cytokine-loaded ISACs may be loaded with IL-12 or IL-15, which has achieved notable success in reversing T cell exhaustion and reinvigorating effector T cells to sustain anti-tumor immunity in response [36].

Moreover, ISACs enable the targeting of immune-cold tumors that lack immune cell infiltration. By stimulating localized immune responses, ISACs transform these immune deserts into immune-active environments. This transformation makes ISACs a promising tool for overcoming resistance to conventional immunotherapies, including immune checkpoint inhibitors. Furthermore, their therapeutic efficacy is enhanced when used in combination with anti-PD-1 therapies, as demonstrated in various preclinical models [33].

### 3.2. BsAbs

BsAbs are a groundbreaking category of antibody-based drugs, capable of binding two distinct antigens simultaneously, usually one tumor antigen and one immune effector receptor, such as CD3 on T cells. This dual-targeting mechanism connects T cells to tumor cells, facilitating tumor lysis. BsAbs can also target inhibitory and stimulatory pathways in the immune system [37,38,39].

One of the most successful applications of BsAbs is in hematologic malignancies. These include bispecific T cell engagers (BiTEs), a class of bsAb that targets CD19 on B-cells while simultaneously engaging CD3 on T cells, including blinatumomab. Blinatumomab has demonstrated unprecedented clinical responses in the treatment of B-cell acute lymphoblastic leukemia (ALL), a malignancy typically resistant to conventional therapies. Its ability to induce complete remission in patients, including those with relapsed or refractory ALL, has set it apart as a breakthrough in the treatment of this challenging disease [40]. The FDA’s approval of blinatumomab for the treatment of ALL marked a significant milestone in immuno-oncology, validating the potential of BsAbs in treating hematologic cancers. Moreover, the success of blinatumomab has spurred ongoing clinical trials exploring the use of BiTEs in other cancers, including non-Hodgkin lymphoma, and expanding the therapeutic applications of BsAbs to solid tumors as well.

Another innovative application involves BsAbs designed to modulate immune checkpoint pathways. For instance, PD-L1/4-1BB BsAbs not only block the interaction of PD-L1 to relieve the inhibition of T cells but also enhance the interaction of 4-1BB, a co-stimulatory receptor that promotes T cell activation and persistence. Such dual-targeting has emerged with encouraging preclinical efficacy in the stimulation of anti-tumor immunity and inducing tumor regression [38]. Furthermore, the combination of immune checkpoint blockade and co-stimulation could address the issue of T cell exhaustion, a common barrier to long-term efficacy in cancer immunotherapy [41]. Ongoing studies are investigating the use of these BsAbs, with early results showing promise in enhancing T cell-mediated anti-tumor responses with various cancers, including melanoma and non-small cell lung cancer [42].

BsAbs are continuously being engineered by new technologies to improve their pharmacokinetics while reducing immunogenicity. Current research is oriented to extend their applications to more types of cancer and to exploit their full potential in combination therapies to maximize clinical benefit.

### 3.3. Checkpoint Blockade Enhancers

Immune checkpoint blockade enhancers represent a new class of therapeutics aimed at enhancing the efficacy of immune checkpoint inhibitors, including anti-CTLA-4 and anti-PD-1/PD-L1 therapies. These targeted enhancers act by targeting the suppressive immune cell populations, including Treg cells and MDSCs, which play a pivotal role in fostering immunosuppression within the tumor microenvironment [43]. By depleting or reprogramming these suppressive cells, immune checkpoint blockade enhancers rejuvenate anti-tumor immune responses and overcome resistance to immune checkpoint inhibitors (ICIs).

Anti-CTLA-4 and anti-PD-1 therapies combined with Treg-depleting mAbs have shown improved efficacy in solid tumors [44]. Furthermore, bispecific checkpoint inhibitors further increase the armamentarium of immune checkpoint blockades, possibly targeting two distinct pathways simultaneously. Examples include bispecific antibodies targeting both TGF-β and PD-L1, inhibiting TGF-β-induced immunosuppression, and restoring T cell activity via PD-L1 blockade. Preclinical testing has shown such a dual-targeting approach induces significant tumor regressions and reinvigorates immune responses in immune-cold tumors [45]. Key findings from a recent study further support the utility of innovative checkpoint blockade enhancers. A bifunctional fusion protein, LH01, composed of an anti-PD-L1 antibody fused to an IL-15/IL-15 receptor α-sushi domain complex was shown to overcome resistance to PD-L1 blockade in preclinical models. This demonstrated the ability to reprogram the TME by enhancing CD8^+^ tumor-infiltrating lymphocytes (TILs), and promoting Th1-type cytokine activity [46].

Beyond depleting suppressive cell populations, immune checkpoint blockade enhancers reprogram the TME to promote pro-inflammatory signaling and enhance antigen presentation. This allows for establishing an immune-active environment that synergizes with ICIs to enhance anti-tumor responses. Combination therapies incorporating checkpoint blockade enhancers with cytokine therapies, T cell agonists, or cancer vaccines are under active investigation and hold great promise in overcoming resistance mechanisms in hard-to-treat cancers. Future research will continue to optimize these strategies and define new targets, further broadening the landscape of checkpoint blockade enhancers to improve outcomes in cancer patients.

## 4. Current Progress and Clinical Applications of ABIs

ABIs development has been fast-tracked by significant preclinical studies, clinical trials, and combination therapy strategies. These efforts have continued to improve our understanding of immune modulation within the TME and the therapeutic potential of ABIs.

### 4.1. Preclinical Studies

Preclinical data have already provided convincing evidence of the potency of ISACs and BsAbs in reprogramming the TME and improving anti-tumor immune responses. ISACs conjugated with TLR7/8/9 agonists have shown the ability to reprogram tumor-associated macrophages and activate dendritic cells, resulting in robust T cell-mediated tumor regression in melanoma and breast cancer models [33,47]. Efforts to optimize ISAC designs have yielded promising results, particularly in improving linker chemistry. These optimizations enhanced payload stability, enabled localized immune activation, minimized off-target effects, and achieved complete tumor eradication in preclinical lymphoma models [37].

Additionally, ISACs delivering STING agonists have demonstrated the ability to reprogram the TME. By converting M2-like macrophages into pro-inflammatory M1-like macrophages, these ISACs enhanced phagocytosis and cytotoxic T cell activation, resulting in significant tumor regression in triple-negative breast cancer (TNBC) models [48].

Similarly, BsAbs targeting HER2 and CD3 have been demonstrated to induce tumor-specific cytotoxic T cell activity in HER2-positive breast cancer mouse models, significantly reducing tumor burden while maintaining minimal systemic toxicity [49]. Further advancements include anti-TGF-β/PD-L1 bispecific antibodies, which effectively depleted Tregs and enhanced CD8^+^ T cell infiltration in non-inflamed tumors, resulting in substantial tumor growth inhibition [50].

In hematologic malignancies, CD19/CD3 bispecific antibodies have shown significant preclinical efficacy. For example, these antibodies effectively redirected T cell cytotoxicity against tumor cells, demonstrating durable tumor regression and prolonged survival in preclinical models of diffuse large B-cell lymphoma (DLBCL) and ALL [51].

Preclinical studies of ABIs have provided valuable insights into their therapeutic potential in cancer immunotherapy. These investigations have focused on optimizing antibody engineering, evaluating immune activation, and addressing challenges related to immune evasion and toxicity.

### 4.2. Clinical Trials

Clinical trials of ABIs are advancing rapidly, with several promising candidates demonstrating efficacy and safety across different types of cancer. Mosunetuzumab, a CD20/CD3 bispecific antibody, stands out as a representative example. By targeting CD20 on B-cells and CD3 on T cells, Mosunetuzumab efficiently redirects cytotoxic T cells to CD20-positive tumor cells, leading to precise tumor cell lysis with minimal damage to healthy tissues [52]. In a Phase II clinical trial (NCT03677154) for relapsed or refractory non-Hodgkin’s lymphoma (NHL), the drug achieved an overall response rate (ORR) of 60%, with 43% of patients achieving complete remission (CR) [52]. These responses were durable, with a median progression-free survival (PFS) of 17.5 months among responders, and the safety profile was favorable, showing a low incidence of cytokine release syndrome (CRS). Building on this success, follow-up studies are exploring combinations of Mosunetuzumab with checkpoint inhibitors, such as anti-PD-1 antibodies, to enhance its therapeutic potential in NHL and other CD20-positive hematologic malignancies like diffuse large B-cell lymphoma (DLBCL). Preclinical findings indicate that such combinations may synergistically enhance T cell activation and mitigate immune evasion in the tumor microenvironment [53]. Beyond Mosunetuzumab, other ABIs are showing promising results in clinical trials (Table 1).

### 4.3. Combination Therapies

Combination therapies involving ABIs have shown promising potential in enhancing anti-tumor efficacy through synergistic mechanisms. Bispecific antibodies have shown potential when combined with other treatment modalities. Radiation therapy combined with CD20/CD3 bispecific antibodies improved immune priming and prolonged survival in preclinical models of B-cell lymphoma, suggesting that localized tumor cell destruction can enhance the efficacy of immune-targeting agents [63]. Similarly, combining small-molecule VEGF inhibitors with HER2/PD-L1 bispecific antibodies in ovarian cancer models effectively controlled angiogenesis while reactivating cytotoxic T cells, highlighting the potential of targeting multiple pathways simultaneously [64].

Moreover, ISACs have demonstrated improved efficacy when used in combination with chemotherapy. In triple-negative breast cancer models, ISACs combined with chemotherapy agents increased tumor immunogenicity and enhanced anti-tumor responses, further supporting the rationale for integrating immunotherapy with traditional treatment modalities [65].

## 5. Emerging Trends and Future Directions in Cancer Immunotherapy

The rapid progress in next-generation antibodies and AI-driven approaches highlights the potential to address major challenges in cancer therapy, such as low clinical response, immune resistance, and off-target toxicity. Bi-or multi-specific antibodies are poised to become the cornerstone of future cancer immunotherapy, with multiple candidates already entering clinical trials. Similarly, AI-driven drug discovery transforms the field by enabling personalized medicine approaches and optimizing therapeutic design. Moving forward, interdisciplinary collaboration between computational scientists and immunologists will be critical to harness the potential of these innovations fully, ultimately advancing cancer treatment paradigms and improving patient survival outcomes.

### 5.1. Next-Generation Drugs

The future of cancer immunotherapy relies on the development of next-generation ABI therapies with the potential to transcend the limitations of current treatments [66]. These innovative strategies place their major focus on the resolution of crucial obstacles that involve immune evasion, treatment resistance, and off-target toxicity while unleashing the full potential of the immune system.

One of the more highly awaited innovations is the design of multi-specific antibodies that target more than one antigen or immune pathway simultaneously. In contrast to what has been carried out thus far, a future multifunctional antibody could seamlessly integrate tumor targeting, immune activation, and microenvironment modulation in one molecule. The main areas of development concern trispecific antibodies that, by adding to the binding with the tumor antigen, introduce recruitment of effector cells, such as T cells via CD3, and co-stimulatory signal activation, for example, CD38 and CD28 [67]. Such next-generation constructs could represent, in this context, an effective strategy in the effort against immune evasion mechanisms and/or in immune-cold, resistant tumors. While preclinical studies have shown great promise, further optimization for stability, manufacturability, and precision is needed before these may see broader clinical application.

In this regard, engineered cytokine–antibody hybrids may eventually redefine the landscape of tumor-specific immune stimulation. These could be a game-changer in immunotherapy by marrying the targeting precision of monoclonal antibodies with the immune-enhancing properties of cytokines such as IL-12 or IL-15 [68]. Moving forward, research is focused on optimizing cytokine stability and tumor-specific delivery to maximize efficacy and minimize adverse effects.

Next-generation therapy goes beyond key single innovations. Multifunctional antibodies, cytokine–antibody hybrids, and ISACs incorporated into personalized combination regimens hold great promise for more durable responses even in cold cancers. Future advances will depend on how successfully main challenges, including manufacturing complexity, immune-related adverse effects, and diversity in patient immune phenotypes, are being overcome.

### 5.2. AI-Driven Precision Medicine

AI is revolutionizing the design and development of antibody-based drugs, accelerating progress in several key areas. The rise of AI, especially Alphafold2, has greatly facilitated the development of antibody drugs. These tools can very accurately design antibody structures, identify targets, and predict efficacy, toxicity, and off-target effects, allowing researchers to prioritize the most promising clinical trial candidates. AI in the development of ABIs evolves the field by allowing rapid, data-driven optimization of structures, functionalities, and therapeutic potentials. AI-powered design platforms are now rapidly accelerating the construction of antibodies, linkers, and immune-stimulatory payloads, addressing challenges such as tumor immune evasion, off-target effects, and systemic toxicity.

Furthermore, AI can predict the stability, binding affinity, and pharmacokinetic properties of certain critical parameters for ABIs through machine learning with big biological datasets. By this, such predictive capabilities obviously reduce the length of trial-and-error experiment time along with increasing the accuracy of the design of antibodies. For instance, AI tools provide the best possible antigen-binding domain to enable such modifications to monoclonal and bispecific antibodies, conferring better tumor specificity with decreased off-target toxicities. In one such example, AI-assisted algorithms designed bispecific antibodies that maximize T cell engagement and minimize unintended interactions to ensure effective immune activation against tumor cells [69].

AI also plays a critical role in the optimization of linkers that connect antibodies to immune-stimulatory payloads or cytotoxic agents. AI can predict how different linker chemistries may affect drug efficacy and safety through the analysis of molecular interactions and stability of linkers. This can result in the development of ISACs, where AI-designed linkers improve payload delivery and activation specifically within the TME. AI-driven algorithms are enabling the discovery of novel therapeutic targets by integrating genomic, proteomic, and transcriptomic datasets. These algorithms identify tumor-specific antigens and immune-suppressive pathways that are not easily accessible using traditional approaches. For instance, AI has identified unique biomarkers in triple-negative breast cancer that are being used to design targeted bispecific antibodies. Such advancements offer new possibilities for treating refractory cancers and expanding the scope of immunotherapy [70].

Looking ahead, AI has the potential to further revolutionize ABI development through enabling automated, end-to-end workflows that integrate target identification, drug design, and preclinical optimization. This interplay of AI with experimental validation will, therefore, be able to realize new opportunities in the design of multifunctional ABIs such as trispecific antibodies and highly specific cytokine–antibody hybrids, thus ushering in a new era of personalized cancer immunotherapy.

## 6. Conclusions

As a pivotal innovation in cancer immunotherapy, ABIs hold great potential for offering a novel approach to orchestrating the TME. They have demonstrated significant efficacy in both preclinical and clinical settings, with a strong focus on targeting immune-suppressive cells, alleviating T cell exhaustion, and delivering immune-stimulatory payloads.

The integration of AI into the development pipeline has further revolutionized ABI research, streamlining the identification of novel therapeutic targets, optimizing antibody structures, and improving preclinical validation. AI-driven tools have facilitated the identification of novel targets, optimized drug design, and improved preclinical validation, enabling precision medicine tailored to individual patient profiles. However, challenges such as immune resistance and toxicity persist, and future research should prioritize the exploration of next-generation antibody designs, combination therapies, and AI-guided strategies to broaden the therapeutic impact of these innovative drugs.

Future research could prioritize AI-guided strategies to enhance the therapeutic impact of ABIs. Integrating AI with multi-omics data analysis, such as transcriptomics and proteomics, can provide deeper insights into tumor immune interactions, leading to more effective biomarker-driven immunotherapies. Additionally, AI-powered simulations can refine combination therapy strategies by predicting synergistic effects between ABIs and other immunotherapies, such as checkpoint inhibitors or CAR-T cells. Advancing these interdisciplinary approaches will be crucial in overcoming current limitations and driving the next generation of ABIs toward broader clinical applications.

By leveraging AI-driven innovations, next-generation ABIs could be designed to exhibit enhanced specificity, reduced immunogenicity, and improved therapeutic efficacy. The continued integration of computational methods with experimental immunotherapy research holds great promise for the development of more personalized and effective treatments for more refractory and drug-resistant cancers.

## Figures and Tables

**Table 1:** Representative ABIs in clinical trials and being marketed.

Drug	Target	Mechanism of Action	Development Stage	Clinical Trial Number	References
Zanidatamab	HER2/CD3 Bispecific Antibody	Redirects T cell cytotoxicity to HER2+ tumor cells	Phase II	NCT04276493	[54]
BDC-1001	HER2-targeted ISAC	Combines HER2-specific antibody with TLR7/8 agonist	Phase I/II	NCT04278144	[55]
REGN5458	BCMA/CD3 Bispecific Antibody	Engages T cells for BCMA-positive tumor lysis	Phase I/II	NCT03761108	[56]
PF-07257876	CD47/PD-L1 Bispecific Antibody	Blocks immunosuppressive TGF-β and PD-L1 signaling	Phase I	NCT04975548	[57]
AMG 160	PSMA/CD3 Bispecific Antibody	Redirects T cells to PSMA-positive tumor cells	Phase I	NCT03792841	[58]
TAK-500	CCR2-targeted ISAC	Targeting the STING pathway and CCR2 expressing myeloid cells	Phase I	NCT05070247	[59]
ARX-788	HER2-targeted ISAC	Combines HER2-specific antibody with TLR7 agonis	Phase I/II	NCT04829604 NCT03255070 NCT05018676	[60]
Blinatumomab	CD19/CD3 Bispecific Antibody	Engages T cells to target CD19-positive B-cell malignancies	Marketed	NCT02013167	[61]
Epcoritamab	CD20/CD3 Bispecific Antibody	Engages T cells to target CD20-positive B-cell	Marketed	NCT04663347	[62]

## Data Availability

No new data were created or analyzed in this study. Data sharing is not applicable to this article.

## Abbreviations

The following abbreviations are used in this manuscript:

TME	Tumor Microenvironment
Treg	Regulatory T cell
MDSC	Myeloid-Derived Suppressor Cell
ISAC	Immune-Stimulating Antibody Conjugate
AI	Artificial Intelligence
TAM	Tumor-associated macrophage
CTL	Cytotoxic T cells
NK	Natural Killer
ROS	Reactive Oxygen Species
IDO	Indoleamine 2,3-dioxygenase
ABI	Antibody-based immune-stimulating drug
BsAbs	Bispecific antibodies
BiTEs	Bispecific T cell engagers
ICIs	Immune checkpoint inhibitors
MAGE	Melanoma-associated antigen
NHL	non-Hodgkin’s lymphoma
ORR	Overall response rate
CR	Complete remission
PFS	Progression-free survival
CRS	Cytokine release syndrome
DLBCL	Diffuse large B-cell lymphoma.
ADCC	Antibody-dependent cellular cytotoxicity
CDC	Complement-dependent cytotoxicity
